# Behind the Procedure: Understanding Complications in Anterior Cervical Surgery

**DOI:** 10.7759/cureus.107395

**Published:** 2026-04-20

**Authors:** Hussam Abu Nowar, Wesam Khraisat, Mu'taz Halasah, Laith Alomari, Osama Alabadi, Khalid Abu-Rumman

**Affiliations:** 1 Neurosurgery Division, King Hussein Medical Center - Royal Medical Services, Amman, JOR; 2 Anaesthesia Division, King Hussein Medical Center - Royal Medical Services, Amman, JOR; 3 Radiology Division, King Hussein Medical Center - Royal Medical Services, Amman, JOR

**Keywords:** acdf, anterior cervical discectomy and fusion, cervical radiculopathy, complications, degenerative cervical disc disease, fusion rate, surgical outcomes

## Abstract

Background: Degenerative cervical disc disease (DCDD) is a frequent cause of neck pain, radiculopathy, and disability in adults. Anterior cervical discectomy and fusion (ACDF) is widely used due to its favorable outcomes and high fusion rates but carries potential perioperative and postoperative complications that require systematic evaluation.

Objectives: This study aims primarily to evaluate complication rates and patterns following ACDF and, secondarily, to assess radiographic fusion outcomes in a tertiary referral center.

Methods and patients: We conducted a retrospective review of adult patients undergoing ACDF for DCDD at King Hussein Medical Center - Royal Medical Services, Amman, Jordan, from 2021 to 2024. Demographics, clinical presentation, operative details, radiological findings, and postoperative complications were analyzed. Patients with at least one-year radiographic follow-up were included in fusion analysis, and fusion was defined as CT trabecular bridging + no motion on flexion-extension.

Results: Final cohort included 284 patients (342 levels). The overall complication rate was 10.56% (n=30), with dysphagia being the most frequent complication (n=6, 2.11%), which lies within the lower range of reported values in the literature (1.7%-9.5%). Radiographic fusion was observed in 98.59% (n=280) of patients with adequate imaging follow-up.

Conclusion: ACDF demonstrated low complication rates and high radiographic fusion in a single-center retrospective cohort. Findings should be interpreted within the limitations of the retrospective design.

## Introduction

Degenerative cervical disc disease (DCDD) is one of the most common clinical scenarios in modern society due to lifestyle changes and technological advances. Individuals with DCDD classically complain of neck pain, radicular pain, and/or sensorimotor symptoms, in combination with other neurological presentations, which are attributed mainly to compression resulting from disc herniation-related local ischemia and cervical nerve root irritation [1-3]. Due to the advancement of early diagnosis and conservative treatment, significant improvements typically happen within the first four to six months after onset in more than 43% of patients [4]. The time to complete recovery ranges from 24 to 36 months and has been reported in approximately 83% of patients in some studies. Individuals who have workers' legal claims showed a worse scenario [5]. Conservative nonsurgical options include immobilization using neck collars, nonsteroidal anti-inflammatory drug (NSAID) administration, muscle relaxant agents, cervical traction, physiotherapy, and epidural steroid injection treatment, separately or in combination, which contribute positively to alleviating symptoms [4,6]. However, in cases where sensorimotor deficits and intractable radiculopathy predominate, surgical treatment is mandatory [7]. Among the most commonly performed surgical treatment options is still the anterior cervical discectomy and interbody fusion (ACDF) [8,9]. This approach has been advocated as a less invasive alternative to the traditional posterior technique since Smith and Robinson reported its first application in 1955 [10]. With reduced morbidity, less interference with the spinal canal, and a higher fusion rate, anterior techniques frequently produce acceptable results [11,12]. While ACDF is associated with a low mortality rate in general, it continues to present a notable morbidity disadvantage, with reported complication rates ranging between 13.2% and 19.3% [2,13]. Therefore, meticulous patient selection remains a critical determining factor of surgical outcomes. This study primarily focuses on analyzing complication patterns associated with ACDF in our institution while secondarily reporting radiographic fusion outcomes. The aim is to provide a realistic complication profile to guide clinical practice rather than to establish comparative effectiveness. However, standardized patient-reported outcome measures, such as the visual analog scale (VAS) or Neck Disability Index, were not included in this study.

## Materials and methods

Ethics

Medical records were retrieved and analyzed from the hospital's electronic database covering a four-year period (2021-2024). This study was approved by the Royal Medical Services Institutional Ethics Board (IRB: 22/14/2025). Given the retrospective design, the requirement for individual patient consent was waived.

Patients

In accordance with the management protocol at our center, all patients underwent an initial comprehensive clinical assessment, followed by radiological evaluation. In the absence of sensorimotor deficits or intractable radiculopathy, a trial of conservative management was initiated. For the final cohort included in this study, surgical intervention was regarded based on the collective clinical and radiological judgments. Diagnosis of degenerative cervical disc disease was based on combined clinical and radiological assessment. MRI evaluation relied on descriptive radiological reporting, including disc herniation, foraminal stenosis, and spinal cord or nerve root compression. No formal grading system was uniformly applied due to retrospective design variability. Imaging follow-up was based on routine outpatient visits and clinical indication. Advanced imaging was not uniformly performed at fixed intervals due to real-world compliance limitations. Inclusion and exclusion criteria are listed in Table 1.

**Table 1:** Inclusion and exclusion criteria * = Ossification of the posterior longitudinal ligament; OPLL = ossified posterior longitudinal ligament

Inclusion Criteria	Exclusion Criteria
Age >18 years	Cervical spine pathology are secondary to oncological conditions
Confirmed cervical disc herniation	Spinal infection or inflammatory process
Failed conservative treatment ≥6 weeks	Prior cervical surgery OPLL*/posterior surgery
Minimum follow-up ≥6 months	Incomplete data

**Figure 1:**
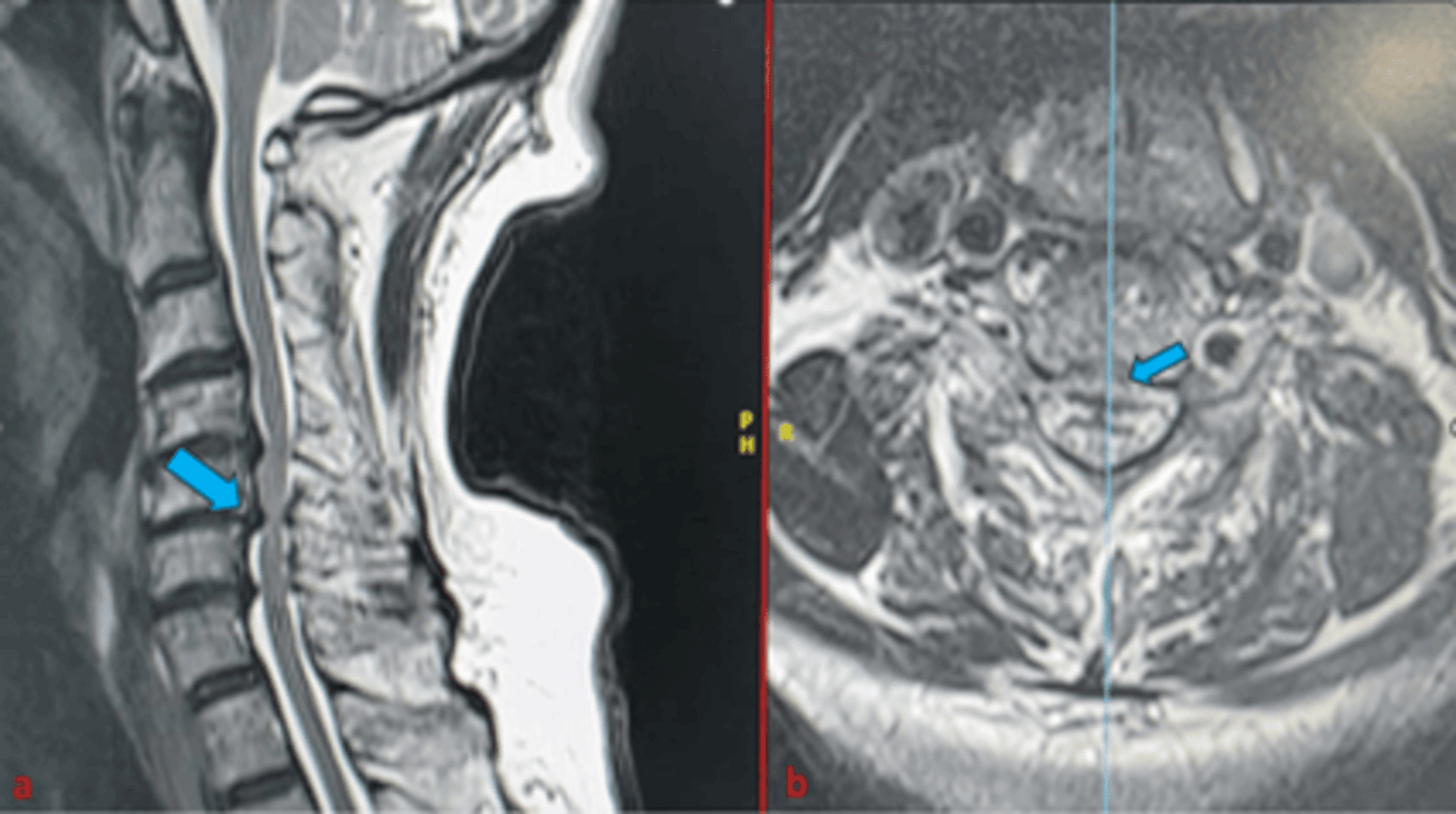
Magnetic resonance imaging scans presenting the preoperative radiological assessment for patients who manifest cervical disc disease signs and symptoms (a) Sagittal and (b) axial. The blue arrow indicates the most prominent degenerative disc herniation.

**Figure 2:**
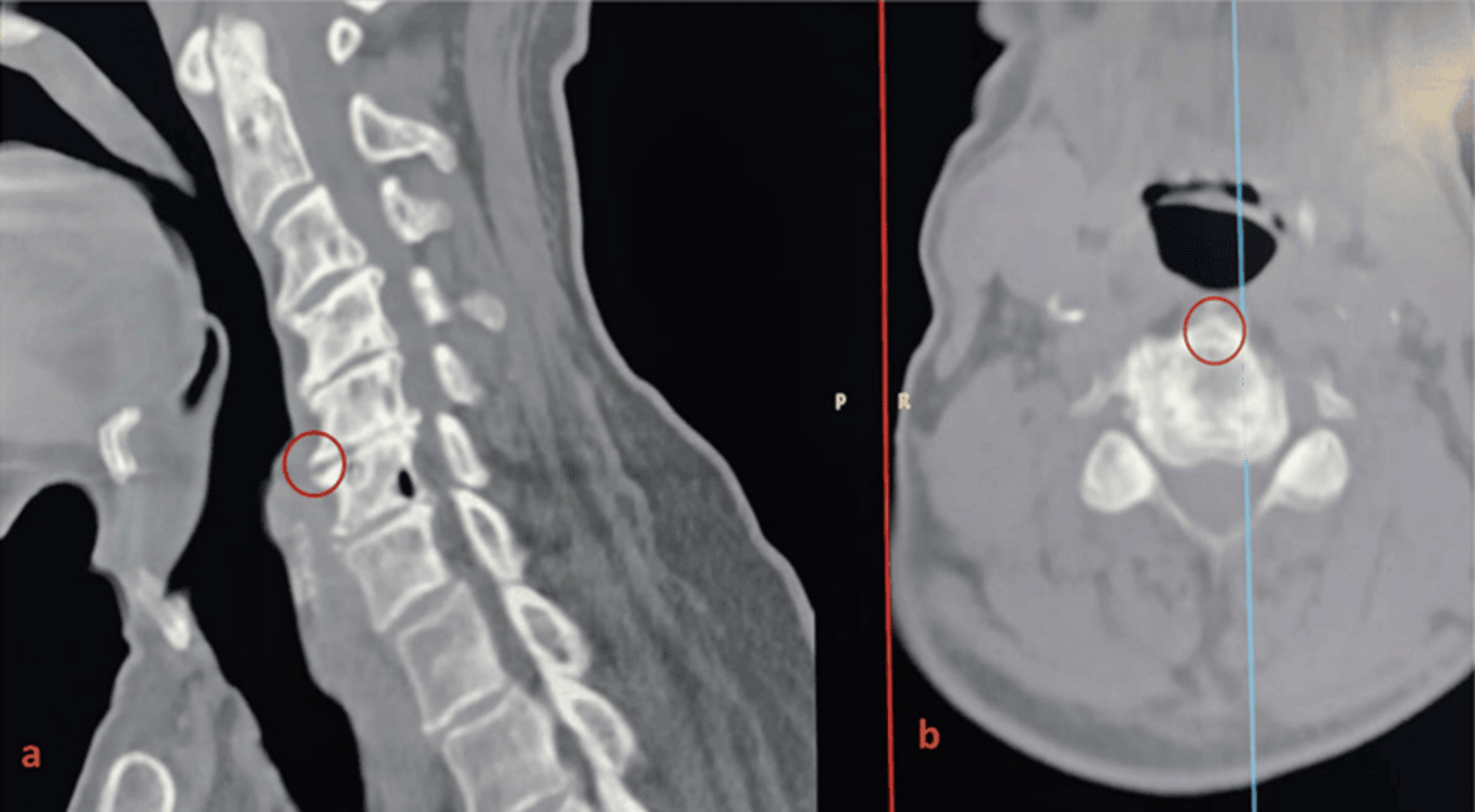
Computed tomography scans disclosing the pre-operative radiological bony detail examination for patients who showed cervical disc disease manifestations (a) Sagittal and (b) axial. The red circle indicates anterior osteophyte.

**Figure 3:**
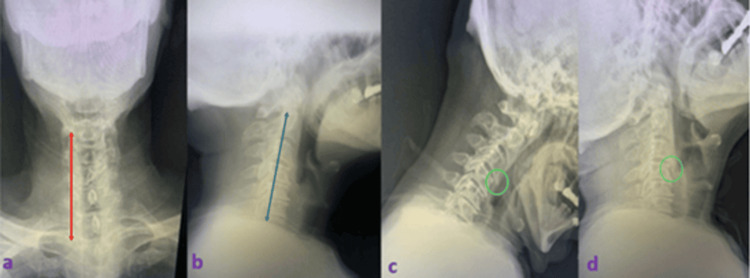
X-ray study showing the primary preoperative radiological assessment to check for dynamic stability in patients who developed cervical disc disease signs and symptoms (a) Antero-posterior, (b) lateral, (c) flexion, and (d) extension images. The orange arrow shows the alignment of vertebrae, the blue arrow indicates the muscle spasm, and the green circle shows minor anterolisthesis.

Detailed documentation was obtained for each patient, encompassing demographic data (age, gender, and body mass index), comorbidities, social habits, and presenting clinical features. A thorough neurological examination was subsequently performed. However, no standardized patient-reported outcome measures, such as the Neck Disability Index or VAS, were collected. Diagnostic confirmation and exclusion of secondary causes of neck and arm pain were accomplished through a series of imaging studies. Fusion was defined as the presence of trabecular bridging bone on CT and/or the absence of motion (<2°) on flexion-extension radiographs, which were obtained after the first year and within the second year postoperatively. Preoperative and postoperative evaluation included magnetic resonance imaging (MRI) (Figure 1), computed tomography (CT) scans (Figure 2), and dynamic radiographs (Figure 3).

Imaging was interpreted by consultant radiologists as part of routine clinical care. In complex cases, multidisciplinary discussion between radiology and surgical teams was undertaken. Formal interobserver variability analysis was not performed.

Study design

This retrospective chart review included all consecutive patients who underwent surgical intervention for cervical disc herniation over the study period, with their corresponding medical records comprehensively reviewed and analyzed in a single referral center. As this study was conducted in a public tertiary hospital, long-term follow-up standardization was limited by patient compliance. However, all included patients had a minimum follow-up of six months and underwent at least one imaging assessment (X-ray and/or CT) at approximately 12 and 24 months (±3 months).

Procedure technical details

After introducing general endotracheal anesthesia, patients were positioned in the supine position, with the neck over the trunk slightly extended, and the target cervical surgical level localization was confirmed intraoperatively using fluoroscopic guidance before incision. The surgical site was meticulously prepared with alcohol and iodine scrub, painted with iodine, and draped in a sterile fashion. A horizontal (neck collar) skin incision, typically 1-2 inches on the right side of the neck within a natural skin crease, was made. The platysma was split along the muscle fibers, and the plane between the sternocleidomastoid and strap muscles was cautiously created using blunt dissection. The plane between the trachea/esophagus medially and the carotid sheath laterally was then dissected, and the prevertebral fascia was dissected to expose the disc space. Fluoroscopy was applied again and reconfirmed at the correct level prior to discectomy to ensure radiological-surgical correlation.

The disc material removal commenced by incising the tough outer layer of an intervertebral disc - the annulus fibrosus - and removing the gel-like center called the nucleus pulposus. Complete disc removal was performed, including curating the cartilage endplates to expose the underlying cortical bone. Dissection continued posteriorly to the posterior longitudinal ligament (PLL), which was gently resected to allow removal of any herniated disc material contributing to spinal cord compression or canal compromise. Partial removal of the uncinate processes was performed in most cases.

Following discectomy, cervical fusion was accomplished by inserting an interbody cage (polyetheretherketone, PEEK) into the disc space to maintain disc height and to facilitate vertebral fusion in all cases. An anterior cervical plate (MaxAn® Anterior Cervical Plate System) was applied in all patients undergoing three-level ACDF and selectively in two-level cases, secured with screws into the adjacent vertebral bodies. To enhance visualization and precision, an operating microscope was utilized in all cases. A soft cervical collar was used for six weeks and discontinued after eight weeks. Physiotherapy began after eight weeks postoperatively with gradual exercises.

Statistical analysis

Microsoft Excel (Microsoft® Corp., Redmond, WA) was used to record all the data, and Statistical Product and Service Solutions (SPSS, version 28.0; IBM SPSS Statistics for Windows, Armonk, NY) was used for analysis. While continuous data were summarized using descriptive statistics methods, such as mean and standard deviation, categorical variables were shown as frequencies and percentages. The independent samples t-test was used to determine the differences in continuous variables between groups. The chi-square test was utilized to assess the correlations between categorical variables. A p-value of less than 0.05 was deemed statistically significant. Comparative statistical analysis was limited to internal group comparisons. External comparisons with previously published studies were performed descriptively using reported ranges from major studies and meta-analyses.

## Results

Of 304 patients screened, out of those, eight patients were disqualified due to additional posterior instrumentation, six patients underwent corpectomy, and six patients were eliminated due to incomplete data. A total of 284 met the inclusion criteria, representing 342 operated levels. Of the final cohort, 62.32% of the patients were males (177/284), exhibiting a mean age at diagnosis of 51.23 ± 12.46 years, with ages spanning 21-69 years. In the female cohort, the mean age at diagnosis was 48.41 ± 8.91 years, with ages spanning 25-64 years, as presented in Table 2. Significant intergroup differences were observed in the number of surgical cases (p < 0.0001), smoking habits (p = 0.035), and peak age (p = 0.002). On the other hand, our study showed several complications. The overall complication rate was 10.56% (n=30), representing patients experiencing at least one complication. Dysphagia was reported as the most common complication, which is within the lower range of complication rates reported in the literature (13.2%-19.3%).

**Table 2:** Demographic and surgery data of the study population χ² = chi-square test for categorical variables, t = independent t-test for continuous variables * indicates statistical significance (p < 0.05).

Parameter	Female	Male	Test	χ²/t value	p value
Number of cases	107	177	χ²	17.25	<0.001
Single-level cases	95 (88.8%)	152 (85.9%)	χ²	0.45	0.50
Two-level cases	12 (11.2%)	25 (14.1%)	χ²	0.45	0.50
Diabetes	28 (26.3%)	46 (25.9%)	χ²	0.01	0.92
Smoker	24 (22.7%)	60 (34.0%)	χ²	4.46	0.035*
Peak age group	40-49	30-39	χ²	-	0.002*
Age (years)	46.67 ± 9.88	47.41 ± 11.68	t	-0.55	0.58
BMI (kg/m²)	27.1 ± 4.8	28.1 ± 5.1	t	-1.67	0.096
Surgery duration (min)	110 ± 40	116 ± 58	t	-1.01	0.31
Blood loss (mL)	173 ± 62	187 ± 156	t	-1.85	0.066
Duration of drainage (days)	1.8 ± 0.4	1.2 ± 0.2	t	1.0	0.31
Mean follow-up (months)	14.16 ± 2.4	14.94 ± 1.99	t	3.65	<0.001

## Discussion

**Figure 4:**
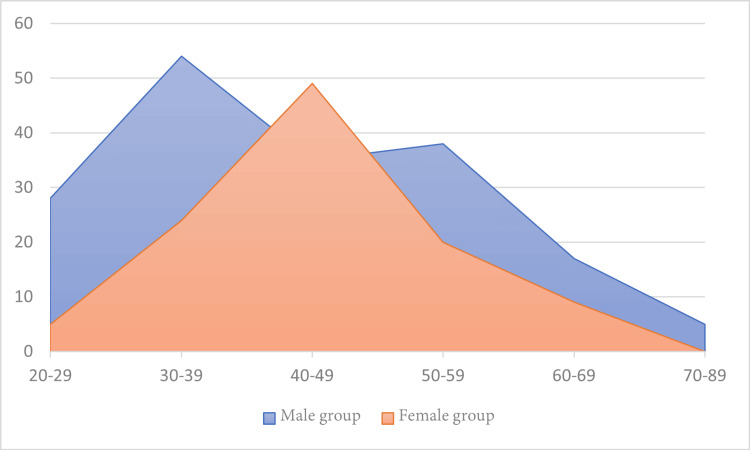
The two subgroups' age distribution Images created by the authors using PowerPoint (Microsoft® Corp., Redmond, WA).

Cervical disc disease represents a prevalent and debilitating spinal condition, with epidemiological data indicating that up to 95% of individuals demonstrate degenerative cervical disc changes by the age of 65 years [14,15]. Occupational hazards exposures involving sustained cervical loading, vibration, or repetitive motion further contribute to accelerated disc degeneration and symptom progression [16]. The highest prevalence of degenerative cervical disc disease is observed in people between 35 and 55 years of age. The condition affects roughly 5.5 individuals per 100,000, with about a quarter of patients eventually requiring surgical intervention due to persistent symptoms or progressive neurological compromise [17,18]. Based on many clinical and epidemiological studies, females appear to have a comparatively lower predisposition to developing degenerative cervical disc disease than males. This sex-based discrepancy has been attributed to differences in occupational exposure, hormonal influences, and lifetime biomechanical loading patterns. Consistent with these findings, our study demonstrated a significantly higher proportion of affected males compared with females (177 vs. 107 cases), and this difference was statistically significant (p < 0.0001), which emphasizes the observation that males are more prone to developing clinically relevant cervical disc degeneration [13,18-20]. Higher rates of cervical spondylotic changes in the general population have been consistently associated with advancing age and gender-related differences [20]. According to our results, in the male cohort, the highest ratio of patients belonged to the 30-39-year age group (30.51%), followed by those aged 50-59 years (21.47%). In contrast, the female cohort showed a different pattern, with the majority of patients falling within the 40-49-year age group (45.79%), followed by the 30-39-year group (22.43%). These distributions are illustrated in Figure 4.

**Figure 5:**
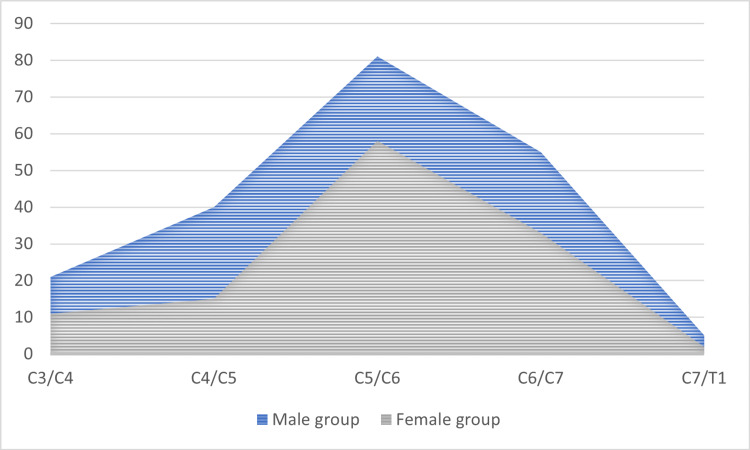
The distribution of cervical discs in the two subgroups

Former studies have repeatedly reported that the most commonly affected cervical disc level is C5-C6, followed by C4-C5, and subsequently C6-C7 [13,21,22]. In comparison, our findings confirmed a similar distribution array, with nearly half of our study population (48.94%) showing degenerative disc disease, which was most frequently observed at C5-C6, with C6-C7 involved in 31% of cases. When placing our study population by sex, a similar pattern was observed in both cohorts. In the male cohort, degenerative involvement at the C5-C6 level was most predominant, accounting for 45.76% of cases, followed by the C6-C7 level (31.07%). In the female cohort, the distribution was comparable, with C5-C6 involvement in 54.21% of cases, followed by C6-C7 involvement in 30.84%, with no statistical difference between the two cohorts (p = 0.164), as illustrated in Figure 5.

**Figure 6:**
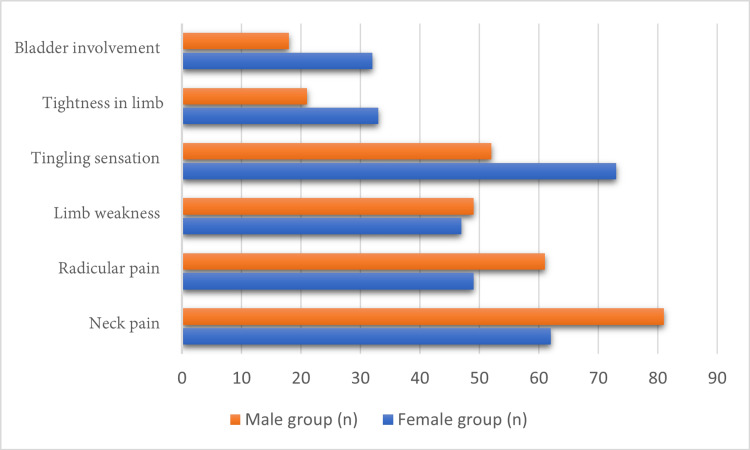
Various clinical manifestations between the two subgroups

Clinical manifestations of cervical disc herniation pathology remain somewhat ambiguous. While neck pain is widely recognized as the cardinal presenting symptom, its precise origin remains to be argued, with ongoing uncertainty regarding the anatomical and physiological sources of pain generation [22-24]. Discogenic pain lacks a totally accepted definition and clear diagnostic criteria, and its role in provoking cervical pain remains controversial despite the frequent association of neck pain with cervical disc herniation in clinical practice [25-29]. In our cohort, however, the most commonly reported symptom was paresthesia, or a tingling sensation, with neck pain reported in second place. Conversely, sphincteric dysfunction represented the least frequent presenting manifestation among cohort patients (Figure 6).

Key diagnostics involve careful musculoskeletal and neurological examinations to distinguish referred pain, true radicular symptoms, and cervical myelopathy, alongside a focused shoulder assessment to rule out primary shoulder pathology [30-32]. For patients with radicular complaints, systematic assessment of upper-limb myotomes and dermatomes is advocated [33,34], and this approach is integrated into our routine clinical practice. When the clinical picture suggests persistent cervical radicular pain lasting more than four to six weeks, an imaging study should be obtained to further evaluate the underlying cause [35].

**Figure 7:**
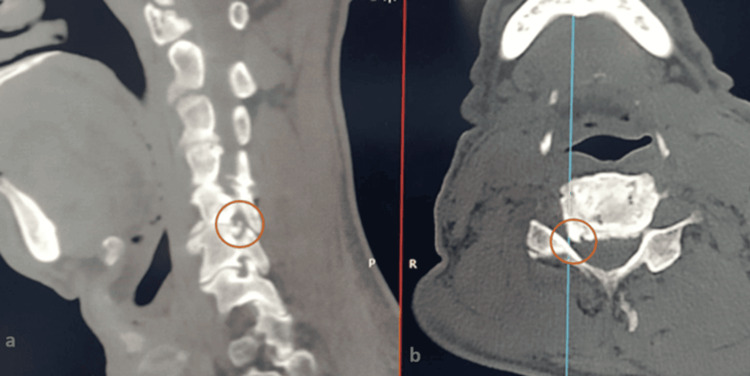
Osteophyte formation is visible in a CT scan of C5/C6 cervical disc degeneration (a) Sagittal and (b) axial slices (marked by the orange circle).

Radiological imaging modalities of the degenerative cervical spine have evolved significantly throughout recent decades of clinical practice. Plain X-rays, including anteroposterior and lateral views, remain the first-line assessment, providing information on overall cervical alignment and identifiable spondylotic changes in facet joints. Lateral dynamic (flexion-extension) views help detect instability not apparent on static images [36]. High-resolution CT with sagittal and coronal reconstructions allows detailed evaluation of bony structures, osteophyte formation, and potential causes of nerve root compression (Figure 7) [37]. MRI is the preferred modality for assessing the spinal cord and soft tissues, especially when neurological findings are inconclusive, offering precise visualization of nerve root pathways [38]. MRI has the highest capability to demonstrate the soft tissue detailed configurations, besides nerve root course from the cord to its exit from the foramen [39]. Meanwhile, electrophysiologic testing can further support the diagnosis of radicular pain [40].

Following diagnostic confirmation and provided that no sensorimotor impairment is present, a six-week course of physiotherapy is recommended, though the exact mechanism of pain relief remains unclear. Non-operative management successfully improves symptoms in 75%-90% of patients with cervical radicular pain [36,37]. Physiotherapy alleviates neck and arm pain, primarily aiming to restore cervical range of motion and strengthen neck muscles [37,41]. Conservative modalities include rest for a brief period, modification of lifestyle, pharmacological agents (nonsteroidal anti-inflammatory drugs, steroids, and/or muscle relaxants), physical therapy, manipulation, injections, and acupuncture [12]. Interventional pain management procedures may offer clinical benefit. Nerve root blocks or epidural steroid injections can provide both diagnostic and therapeutic purposes [12].

For patients who have not responded well to a course of conservative management or who present indications for surgery, such as significant neurological deficits or intractable pain, multiple valid surgical options exist for the treatment of cervical radicular pain [12].

The anterior approach, combined with allograft usage, offers several advantages. It helps restore cervical segmental lordosis, stabilizes the vertebral column, promotes solid fusion, functions as a load-sharing construct, and provides both direct and indirect decompression of the affected nerve roots [42,43]. Historically, excised intervertebral discs were either replaced with autologous bone grafts or left unfilled, a practice traditionally followed after disc excision [44-48]. More recently, surgeons have increasingly relied on allografts or interbody cages to facilitate restoration of normal disc height [49,50].

In routine practice, ACDF is our preferred procedure for degenerative cervical disc disease management. Even with its high success in fusion and alignment restoration, ACDF remains associated with several potential complications [51-53].

The ACDF procedure's morbidity/complication rates ranged from 13.2% to 19.3%, according to many analyses [51-55]. In our cohort, the overall morbidity rate was 10.56%, which falls within the lower range of complication rates reported in the literature (13.2%-19.3%). Similarly, when compared with a previous study conducted at our institution that documented a morbidity rate of 16.56%, no statistically significant difference was detected (p ≈ 0.079) [13].

To assess our effectiveness and safety, we compared each complication we had with the documented literature. While some of these issues may arise during surgery, others may manifest in a late fashion during the postoperative course.

Among the most common postoperative complications following anterior cervical procedures is dysphagia. In our series, it was observed in six patients (2.11%), with all cases resolving spontaneously within two weeks' time. Worldwide reports describe a broad incidence range of 1.7%-9.5% [56-59].Our results fall within the lower spectrum of reported values (1.7%-9.5%).

One of the most serious and deadly side effects that might occur is esophageal perforation, reported in 0.3%-0.9% of cases in the literature [60-62]. Luckily, our cohort did not demonstrate any occurrence of this complication.

Unintended dural breach or tear, although unusual, remains a potentially serious complication. In our cohort, it occurred in a single patient (0.35%). Fortunately, no cerebrospinal fluid (CSF) fistula developed postoperatively, and the case was efficiently managed with primary closure supplemented by a fibrin sealant patch. The patient experienced no further adverse effects. When compared with the reported incidence in the literature (0.24%-1.7%), which may increase substantially in case of multilevel ACDF or in cases of ossified posterior longitudinal ligament (OPLL) [13,63,64], our findings were consistent with reported rates in the literature (0.24%-1.7%).

Recurrent laryngeal nerve (RLN) palsy is a recognized complication of anterior cervical spine surgery. Previous reports suggest that its true incidence is often underreported and may be higher than traditionally assumed [65]. Unilateral RLN palsy typically manifests as dysphonia or hoarseness, whereas bilateral involvement may result in respiratory compromise requiring urgent intervention [66]. RLN injury can result from excessive or prolonged retraction, soft-tissue edema, thermal damage, or increased endotracheal tube cuff pressure. Prevention focuses on meticulous handling of soft tissues by minimizing retraction, maintaining a midline operative corridor, coordinating cuff-pressure adjustments with anesthesia, avoiding unnecessary cautery near the tracheoesophageal groove, and electrophysiology monitoring was advocated to decrease incidence [65,67]. In our cohort, symptomatic RLN palsy occurred in two patients (0.70%). Literature reports an incidence of incidental, asymptomatic preoperative RLN palsy of about 1.6% [13], while postoperative symptomatic RLN palsy after ACDF is reported in the range of 1.1%-3.1% [13,65,66,68]. Our findings were within the lower range of reported values (1.1%-3.1%).

Horner's syndrome is a known, but uncommon, complication associated with anterior approaches to the subaxial cervical spine [69]. It is characterized by the classic triad of ipsilateral pupillary miosis, partial ptosis, and facial anhidrosis, originating from cervical sympathetic chain damage. Horner's syndrome rarely results in considerable functional impairment, but patients may be concerned about its cosmetic effects [70].

The pathophysiology is most commonly attributed to injury of the sympathetic trunk, which lies in close proximity to the longus colli muscle during surgical exposure. Accordingly, preventive strategies focus on maintaining a midline operative plane, minimizing retraction time and pressure, and avoiding unnecessary manipulation.

Reported incidence in the literature is exceptionally low, ranging from 0.02% to 4.0% [52,53,71,72]. In our cohort, one patient (0.35%) developed Horner's syndrome. Despite management with corticosteroids and supportive speech therapy, the patient demonstrated incomplete recovery.

Any surgical procedure, including anterior cervical spine surgery, is known to result in both superficial and deep operative site infections. Pathogenesis is commonly linked to bacterial contamination during surgery, hematoma formation, prolonged operative time, or patient-related risk factors, such as diabetes, smoking, and poor nutritional status, with reported incidences ranging from 0.9% to 1.6% [72,73]. In our series, five patients (1.76%) developed superficial wound infections. All cases were managed successfully with local wound care and empirical antibiotic therapy, and none progressed to deep infection or required surgical intervention.

Preventive measures include strict adherence to aseptic technique, appropriate perioperative antibiotic prophylaxis, minimizing operative time, meticulous hemostasis to reduce hematoma risk, and optimization of modifiable patient comorbidities preoperatively. Our observed incidence is comparable to reported rates (0.9%-1.6%).

Despite being rare after ACDF, postoperative hematoma is a potentially fatal consequence. To avoid airway compromise, early identification and quick surgical evacuation are crucial. The range of reported occurrence in published articles is 0.2%-2.4% [12,51,68,71,74]. Hematomas may develop either acutely or in a delayed fashion, with most delayed cases presenting approximately six days after surgery [75,76]. Meticulous intraoperative hemostasis and the use of closed-suction drains are regarded as key preventive strategies [74].

In our study, postoperative wound hematoma requiring surgical evacuation was observed in two patients (0.70%). This rate was within the lower range of reported values in the literature (0.2%-2.4%).

Adjacent intervertebral level disc degeneration is relatively a common medium to long-term postoperative consequence, resulting from increased mechanical stress and instability loads to the nearby intervertebral discs, as well as segmental changes in the biomechanics [76-79]. It had a substantial correlation with the procedure, even though it was not one of the ACDF direct complications. According to recent studies, the incidence of neighboring segment degeneration was approximately 12.2% following ACDF and increased to 25% following a second ACDF [68,71,80,81]. According to other research, 9% of neighboring level diseases appeared six years after ACDF, and 5.9% of these finally needed surgery. This translates to an estimated 0.9% annual rate of degeneration [82,83]. Our review identified six cases (2.11%) of adjacent-level disease within three years of primary surgery; our results are within the lower range of reported values. Morbidity rates are presented in Table 3.

**Table 3:** Postoperative complications following anterior cervical discectomy and fusion (ACDF) Reported ranges are derived from previously published studies and meta-analyses [13,51-55,56-59,60-68,71-74,76-81,84-88]. No direct statistical comparison was performed due to heterogeneity across studies. RLN: Recurrent laryngeal nerve

Complication	Cases (n)	%	Reported Range (Literature)
Dysphagia	6	2.11	1.7-9.5%
Esophageal perforation	0	0.0	0.3-0.9%
Dural tear	1	0.35	0.24-1.7%
RLN palsy	2	0.70	1.1-3.1%
Horner’s syndrome	1	0.35	0.02-4.0%
Infection	5	1.76	0.9-1.6%
Hematoma	2	0.70	0.2-2.4%
Adjacent segment disease	6	2.11	~9% (Long-term)
Pseudoarthrosis	4	1.41	Up to 9.1%
Cage failure	3	1.06	3-42%
Overall	30	10.56	13.2-19.3%

The primary goal following neural and spinal decompression in ACDF is to accomplish a robust and rigid intervertebral fusion. According to published research, fusion rates for one- to two-level ACDF were greater after one-level compared to two-level ACDF, and they were also higher when employing autograft [84,85]. The presence of trabecular bridging bone growth between the cage and vertebral endplate, as shown by a computed tomography scan, and the absence of motion verified by a dynamic cervical X-ray of the fused levels are the requirements to enable bone fusion [86]. Research revealed fusion rates ranging from 90.1% to 100% [85-88]. Our series demonstrated a fusion rate of 98.59%, while pseudoarthrosis was significantly lower, at a rate of 1.41%, confirmed in four cases, which is lower than commonly reported rates in the literature.

Mechanical failure of interbody cages is an uncommon but clinically critical complication following ACDF. It may manifest as cage subsidence, migration, or collapse, leading to loss of segmental height, kyphotic deformity, persistent radicular symptoms, or delayed fusion [86-88]. Several predisposing mechanisms have been proposed, including inadequate endplate preparation, excessive distraction, osteoporosis, or high biomechanical loads transmitted across the operated segment [87,88]. Cage subsidence remains the most frequently reported form of mechanical failure, with incidence rates ranging between 19.3% and 42.5% depending on implant design, bone quality, and surgical technique [86]. Proper endplate preparation, appropriate cage size and material selection, and balanced postoperative sagittal alignment are considered essential preventive strategies. In our cohort, mechanical failure occurred in three cases (1.06%), results were within the lower range of reported values.

The overall complication rate revealed in our cohort was 10.56%; no mortality was reported. Higher complication rates were observed among older patients, smokers, and those undergoing multilevel procedures; however, these observations are descriptive and not based on adjusted analysis, reflecting well-established risk factors in the literature. Nevertheless, meticulous preoperative assessment, accurate diagnosis, and adherence to consistent surgical principles remain pivotal in minimizing adverse events and optimizing postoperative outcomes.

Although this study provides reliable and clinically meaningful results, it still carries considerable limitations. First, its retrospective, single-center design introduces inherent selection and information bias, limiting the generalizability of the findings. The absence of a control or comparator group restricts the ability to draw causal inferences or compare outcomes with alternative surgical approaches. Additionally, the analysis is primarily descriptive, as no multivariate regression was performed to identify independent predictors of complications, making observed associations exploration rather than definitive. Standardized clinical outcome measures, such as patient-reported outcomes or functional scores, were not included, which limits assessment of overall clinical effectiveness. Radiological evaluation was based on routine clinical reporting without the use of a uniform grading system or formal interobserver agreement analysis, potentially affecting consistency. Furthermore, imaging follow-up was not fully standardized due to the nature of a public healthcare setting and variable patient compliance, introducing potential detection bias. Finally, although all included patients had a minimum follow-up and interval imaging, longer-term outcomes - particularly for multilevel procedures and adjacent segment disease - may not be fully captured. Further prospective, multicenter studies with larger patient populations are warranted to refine management strategies, enhance surgical outcomes, and minimize the incidence of postoperative complications.

## Conclusions

Anterior cervical discectomy and fusion demonstrated generally favorable and clinically meaningful low complication rates in most patients within the reported literature ranges. This study primarily provides a complication-focused analysis, and conclusions regarding effectiveness remain limited by the retrospective design, absence of standardized clinical outcome measures, and lack of comparative groups. Nevertheless, although complications were infrequent, the potential for adverse or disruptive events must still be acknowledged and carefully considered during clinical decision-making.

The study could not identify specific modifiable preoperative risk factors that could potentially reduce or prevent postoperative complications. On the other hand, optimizing patient results and improving quality of life remain strongly dependent on maintaining a high level of clinical awareness, guaranteeing early recognition of symptoms, and providing prompt, appropriate management strategies. These factors play a pivotal role in influencing prognosis and overall treatment success.
